# Design, Synthesis and Biological Evaluation of New 5,5-Diarylhydantoin Derivatives as Selective Cyclooxygenase-2 Inhibitors

**DOI:** 10.3797/scipharm.1104-20

**Published:** 2011-07-25

**Authors:** Afshin Zarghi, Farin Sattary Javid, Razieh Ghodsi, Orkideh G. Dadrass, Bahram Daraei, Mehdi Hedayati

**Affiliations:** 1 Department of Pharmaceutical Chemistry, School of Pharmacy, Shahid Beheshti University of Medical Sciences, Tehran, Iran; 2 Department of Pharmaceutical Chemistry, School of Pharmacy, Azad University, Tehran, Iran; 3 Department of Toxicology, Tarbiat Modarres University, Tehran, Iran; 4 Research Institute for Endocrine Sciences, Shahid Beheshti University (M.C), Tehran, Iran

**Keywords:** 5,5-Diarylhydantoin derivatives, COX-2 inhibition, Molecular modeling studies, SAR

## Abstract

A new group of 5,5-diarylhydantoin derivatives bearing a methylsulfonyl COX-2 pharmacophore at the *para* position of the C-5 phenyl ring were designed and synthesized as selective COX-2 inhibitors. In vitro COX-1/COX-2 inhibition structure-activity relationships identified 5-[4-(methylsulfonyl)phenyl]-5-phenyl-hydantoin (**4**) as a highly potent and selective COX-2 inhibitor (COX-2 IC_50_ = 0.077 μM; selectivity index > 1298). It was more selective than the reference drug celecoxib (COX-2 IC_50_ = 0.060 μM; selectivity index = 405). A molecular modeling study where **4** was docked in the binding site of COX-2 indicated that the *p*-MeSO_2_ COX-2 pharmacophore group on the C-5 phenyl ring is oriented in the vicinity of the COX-2 secondary pocket. The results of this study showed that the type of substituent on the N-3 hydantoin ring substituent is important for COX-2 inhibitory activity.

## Introduction

Non-steroidal anti-inflammatory drugs (NSAIDs) are widely utilized for the treatment of various inflammatory conditions such as rheumatic fever, rheumatoid arthritis and osteoarthritis. These drugs are competitive inhibitors of cyclooxygenase (COX), a rate limiting enzyme that mediates the bioconversion of arachidonic acid to inflammatory prostaglandins (PGs) [1, 2]. COX is a membrane-bound heme protein which exists at least in two different isoforms, COX-1 and COX-2 [3]. COX-1, described as a “housekeeping” enzyme, is normally expressed in the gastrointestinal tract, kidneys and platelets. Under the influence of COX-1, prostaglandins maintain the integrity of the gastric mucosa, mediate normal platelet function and regulate renal blood flow [4]. The isoenzyme COX-2 is primarily associated with inflammation. Cytokines and growth factors increase the expression of COX-2, mainly at inflammatory sites, producing prostaglandins that mediate inflammation, pain and fever [5]. However, because NSAIDs inhibit both isoforms of cyclooxygenase (COX) their use is often accompanied by gastrointestinal side effects and renal function suppression [6]. It is known that selective COX-2 inhibitors can provide anti-inflammatory agents devoid of the undesirable effects associated with classical non selective NSAIDs. Moreover, recent studies indicating the place of COX-2 inhibitors in the prevention of several cancer types such as colon, breast, lung and prostate cancers [7–10] and neurodegenerative diseases such as Parkinson [11] and Alzheimer’s [12] diseases still continues to attract investigations on development of COX-2 inhibitors. 1,2-Diaryl-heterocycles, and other central ring pharmacophore templates, have been extensively studied as the most important selective COX-2 inhibitors. Several series of selective COX-2 inhibitors belonging to the 1,2-diaryl class of compounds containing different heterocyclic and carbocyclic moieties as a central scaffold have been developed. All these tricyclic molecules possess a characteristic sulfonyl group such as a *para*-SO_2_NH_2_ or a *para*-SO_2_Me substituent on one of the phenyl rings which plays an important role on COX-2 selectivity [13]. Celecoxib and rofecoxib are two well-known selective COX-2 inhibitors belong to COXIBs class [14, 15]. However, the market withdrawal of some COXIBs such as rofecoxib due to increase the risk of heart attack and cardiovascular side effects [16] encourages the researchers to explore new selective COX-2 inhibitors to evaluate their effects and may improve the safety profiles. In addition, recent studies have suggested that rofecoxib’s adverse cardiac events may not be a class effect but rather related to its particular chemical structure [17]. For this reason novel scaffolds with high selectivity for COX-2 inhibition need to be found and evaluated for their biological activities. In this regard, several selective COX-2 inhibitors with geminal diaryl groups (see structures **A**, **B**) have been reported that exhibited considerable potency and selectivity on COX-2 inhibition [18]. As part of our research program, aimed at discovering new selective COX-2 inhibitors, we focused our attention on the synthesis, COX inhibitory and some molecular modeling studies of 5,5-diarylhydantoin derivatives possessing a methylsulfonyl COX-2 pharmacophore at the *para* position of the C-5 phenyl ring. In these designed compounds we utilized geminal diarylhydantoin as new scaffold of COX-2 inhibitors.

## Results and Discussion

The target 5,5-diarylhydantoin derivatives **4–8** were synthesized via the route outlined in Scheme 1. As illustrated in the Scheme 1, 1-[4-(methylthio)phenyl]-2-phenylethanone (**1**) was synthesized by Friedel-Crafts acylation of thioanisole with phenylacetyl chloride (yield 75%) [19]. Oxidation of **1** with selenium dioxide in dioxane-water under reflux condition gave 1-[4-(methylthio)phenyl]-2-phenylethane-1,2-dione (**2**) (yield 92%) [19]. Reaction of **2** with urea under alkaline condition afforded 5-[4-(methylsulfanyl)phenyl]-5-phenylhydantoin (**3**, yield: 37%) [20]. Oxidation of **3** with oxone in THF-water gave the oxidized methyl-sulfonyl compound **4** (yield 67%) [21].The alkylated hydantoin derivatives **5–8** were prepared using an appropriate alkyl halide in alkaline ethanol (yield: 27–54%) [22]. The purity of all products was determined by thin layer chromatography using several solvent systems of different polarity. All compounds were pure and stable. The compounds were characterized by NMR, infrared, mass spectrometry and CHN analysis.

**Fig. 2.**
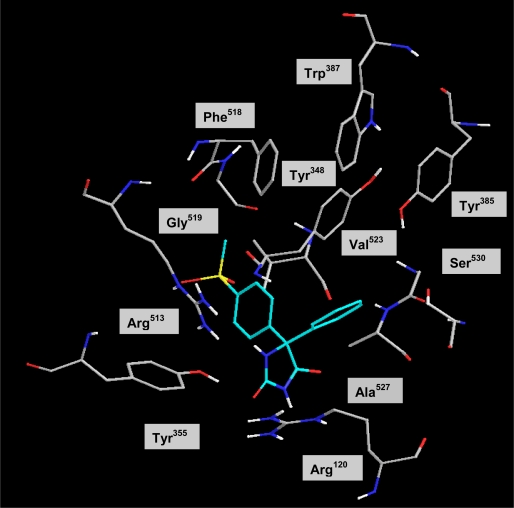
Docking of **4** in the active site of murine COX-2.

**Fig. 3.**
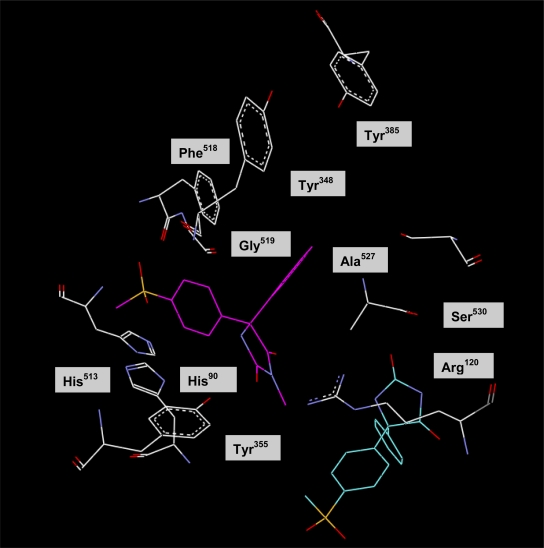
Docking of **4** (in blue) and **5** (in pink) in the active site of murine COX-1.

All of the prepared 5,5-diarylhydantoin derivatives having different substituents at the N-3 hydantoin ring were evaluated to investigate the effect of different alkyls on COX-2 selectivity and potency. The ability of the 5,5-diarylhydantoin **4–8** to inhibit the COX-1 and COX-2 isozymes was determined using chemiluminescent enzyme assays (see enzyme inhibition data in Table 1.) according to our previously reported method [23]. In vitro COX-1/COX-2 inhibition studies showed that all compounds **4–8** were selective inhibitors of the COX-2 isozyme with IC_50_ values in the highly potent 0.077 to 0.171 μM range, and COX-2 selectivity indexes (S.I.) in the 70.2 to > 1298 range. The relative COX-2 selectivity profiles for the 5,5-diarylhydantoin derivatives **4–8**, with respect to the N-3 substituent (R) was H > Me > Et> Allyl > Pr. SAR data (IC_50_ values) acquired by determination of the in vitro ability of the title compounds to inhibit the COX-1 and COX-2 isozymes showed that the COX inhibition is sensitive to the size of substituent at the N-3 hydantoin ring. These data showed that the type of substituent attached to N-3 of hydantoin ring affected selectivity for COX-2 inhibitory activity. Accordingly, compounds having larger groups at the N-3 central ring showed less selectivity for COX-2 isozyme that can be explained by steric parameter. However, among the 5,5-diarylhydantoin derivatives, compound **4** with no substituent at N-3 hydantoin ring exhibited the highest COX-2 inhibitory selectivity (COX-2 IC_50_ = 0.077 μM; SI > 1298) that was more selective than the reference drug celecoxib (COX-2 IC_50_ = 0.060 μM; SI = 405). In addition, our results showed that the unsubstituted compound **4** had significantly higher selectivity index compared with the alkylated analogues **5–8**. This difference is mainly due to poor affinity of compound **4** for COX-1 (COX-1 IC_50_ > 100 μM; SI > 1298) comparison to alkylated hydantoins which showed more activities for COX-1 inhibition. Our in vitro enzyme inhibition data also showed that the size of N-3-alkyl is important for COX-1 inhibitory activity and therefore it can affect the selectivity index. In addition to steric parameter, the unsubstituted hydantoin **4** is more acidic than the alkylated compounds and therefore it can be as keto-enol forms. This effect may cause different tautomer forms for compound **4** and may explain its different interaction in COX-1 active site relative to its 3-alkylated derivatives. Accordingly, the binding interactions of the most potent and selective COX-2 inhibitor compound (**4**) within the COX-2 binding site were investigated. The most stable enzyme-ligand complex of 5-(4-methylsulfonyl) phenyl-5-phenyl-hydantoin (**4**) possessing a MeSO_2_ COX-2 pharmacophore at *para* position of C-5 phenyl ring within the COX-2 binding site (Fig. 2) shows that the *p*-MeSO_2_–phenyl moiety is oriented towards the COX-2 secondary pocket (Arg^513^, Phe^518^ and Val^523^). One of the *O*-atoms of *p*-MeSO_2_ substituent forms a hydrogen binding interaction with amino group of Arg^513^ (distance = 4.6 A^o^) whereas the other *O*-atom is about 3.5 Å away from N*H* of Arg^513^. In addition, a hydrogen bonding interaction can form between the N*H* group at position 3 of hydantoin ring and nitrogen atoms of Arg^120^ (distance = 4.8 Å) which may explain the high potency and selectivity of compound **4**. In this regard, a similar molecular modeling study was performed where compounds **4** and **5** were docked in the COX-1 binding site (Fig. 3) to explain the difference COX-1 activities of compound **4** and alkylated compounds such as **5.** Our results indicated that the most stable enzyme-ligand complex of N-3-methyl-5-(4-(methylsulfonyl)phenyl)-5-phenylhydantoin **(5)** well docked in the COX-1 active site whereas the unsubstituted compound **4** was not docked into the active site of COX-1 as well as its alkylated analogue **5**. These results can facilitate the interpretation of in vitro enzyme inhibition structure-activity data.

## Conlcusion

This study indicates that (i) the hydantoin ring is a suitable scaffold to design COX-2 inhibitors, (ii) COX-2 selectivity index is sensitive to the type of N-3 hydantoin substituent, and (iii) 5-[4-(methylsulfonyl)phenyl]-5-phenyl-hydantoin (**4**) exhibited high COX-2 inhibitory potency and selectivity.

## Experimental

### Chemistry

All chemicals and solvents used in this study were purchased from Merck AG and Aldrich Chemical. Melting points were determined with a Thomas–Hoover capillary apparatus. Infrared spectra were acquired using a Perkin Elmer Model 1420 spectrometer. A Bruker FT-500 MHz instrument (Bruker Biosciences, USA) was used to acquire ^1^HNMR spectra with TMS as internal standard. Chloroform-D and DMSO-D_6_ were used as solvents. Coupling constant (*J*) values are estimated in hertz (Hz) and spin multiples are given as s (singlet), d (double), t (triplet), q (quartet), m (multiplet), and br (broad). Low-resolution mass spectra were acquired with a MAT CH5/DF (Finnigan) mass spectrometer that was coupled online to a Data General DS 50 data system. Electron-impact ionization was performed at an ionizing energy of 70 eV with a source temperature of 250 °C. A 6410 Agilent LCMS triple quadrupole mass spectrometer (LCMS) with an electrospray ionization (ESI) interface was also used for molecular weight measurement. Microanalyses, determined for C, H, and N were within ± 0.4% of theoretical values.

#### 1-[4-(Methylsulfanyl)phenyl]-2-phenylethanone (**1**)

Phenacetyl chloride (4 ml, 30.2 mmol) was added to a suspension of anhydrous aluminum chloride (4.2 g, 31.6 mmol) in dry dichloromethane (50 ml) under argon atmosphere at 0 °C. After stirring the reaction mixture for 30 min, thioanisole (3 ml, 24 mmol) was slowly added during 15 min. After keeping the mixture at this temperature for 2 hrs, it was allowed to stir at room temperature overnight. Then the mixture was poured over crushed ice and extracted with dichloromethane (3×50 ml), and the combined organic layer after washing with water was dried by anhydrous MgSO_4_ and evaporated. The solid product was recrystalized in ethanol. Yield: 75%; mp: 97–98 °C; IR (KBr): ν (cm^−1^) 1685 (CO), ^1^HNMR (CDCl_3_): δ (ppm) 2.52 (s, 3H, SMe), 4.22 (s, 2H, CH_2_), 7.21–7.33 (m, 7H, Phenyl H_2_-H_6_ & 4-methylthiophenyl H_3_ & H_5_), 7.91 (d, 2H, 4-methylthiophenyl H_2_ & H_6_, *J*=8.4 Hz). LC-MS (ESI) m/z: 243.1 (M+1) (100).

#### 1-[4-(Methylsulfanyl)phenyl]-2-phenylethane-1,2-dione (**2**)

Selenium dioxide (2.5 g, 22.5 mmol) was dissolved in a mixture of 1,4-dioxane-water (50 ml, 48:2). The 1,4-dioxane solution of **1** (2.5 g, 10.3 mmol), was added to the reaction mixture and refluxed overnight. The precipitated selenium was filtered off and the filtrate was poured over ice water. After extraction with ethyl acetate (3×50 ml) the combined organic phase was washed with water, dried and evaporated to get yellow solid and crystallized in ethanol. Yield: 92%; mp: 58–59 °C; IR (KBr): ν (cm^−1^) 1675, 1586 (CO); ^1^HNMR (CDCl_3_): δ (ppm) 2.62 (s, 3H, SMe), 7.13–7.42 (m, 7H, Phenyl H_2_-H_6_ & 4-methylthiophenyl H_3_ & H_5_), 8.06 (d, 2H, 4-methylthiophenyl H_2_ & H_6_, *J*=8.3 Hz); LC-MS (ESI) m/z: 257.1 (M+1) (100).

#### 5-[4-(Methylsulfanyl)phenyl]-5-phenylimidazolidine-2,4-dione (5-[4-(Methylthio)phenyl]-5-phenylhydantoin, **3**)

A solution of **2** (1.5 g, 5.8 mmol) and urea (0.4 g, 6.7 mmol) in 30% aqueous sodium hydroxide and ethanol (50 ml) acid was refluxed for 3 hours. The reaction mixture was then cooled and poured into ice-cold water. The mixture was filtered and the filtrate was acidified with concentrated hydrochloric acid, cooled in ice-water and the precipitated product was filtered under vacuum. After washing with water, the product was crystallized in ethyl acetate Yield: 37%; mp: 115–116 °C; IR (KBr): ν (cm^−1^) 3568, 3287 (NH), 1709 (CO), ^1^HNMR (CDCl_3_): δ (ppm) 2.42 (s, 3H, SMe), 7.24–7.32 (m, 7H, Phenyl H_2_-H_6_ & 4-methylthiophenyl H_3_ & H_5_), 7.36 (d, 2H, 4-methylthiophenyl H_2_ & H_6_, *J*=8.2 Hz); LC-MS (ESI) m/z: 299.1 (M+1) (100); Anal. Calcd. for C_16_H_14_N_2_O_2_S: C, 64.41; H, 4.73; N, 9.39. Found: C, 64.62; H, 4.99; N, 9.22.

#### 5-[4-(Methylsulfonyl)phenyl]-5-phenylimidazolidine-2,4-dione (5-[4-(Methylsulfonyl)phenyl]-5-phenylhydantoin, **4**)

0.5 g (1.7 mmol) of hydantoin **3** was dissolved in 10 ml THF and 2.5 g oxone in THF/water was added. The mixture was stirred at room temperature for 4 hours, after evaporation of THF, the residue was extracted with ethyl acetate and dried with sodium sulfate and then evaporated. The product was recrystallized in ethanol. Yield: 67%; mp: 223–224 °C; IR (KBr): ν (cm^−1^) 3210, 3100 (NH), 1727, 1706 (CO),1307, 1154 (SO_2_); ^1^HNMR (CDCl_3_): δ (ppm) 3.18 (s, 3H, SO_2_Me), 7.29–7.40 (m, 5H, Phenyl), 7.59 (d, 2H, 4-methylsufonylphenyl H_2_ & H_6_, *J*=8.4 Hz), 7.97 (d, 2H, 4-methylsufonylphenyl H_3_ & H_5_, *J*=8.4 Hz); 9.42 (s, 1H, NH), 11.23 (s, 1H, NH); ^13^C-NMR (CDCl_3_): δ 44.1, 71.1, 126.1, 128.3, 128.7, 129.4, 129.6, 136.2, 139.8, 144.9, 157.0, 174.6; LC-MS (ESI) m/z: 331.1 (M+1) (100); Anal. Calcd. for C_16_H_14_N_2_O_4_S: C, 58.17; H, 4.27; N, 8.48. Found: C, 58.33; H, 4.59; N, 8.32.

#### General Procedure for the Synthesis of N-3-alkylated hydantoins **5–8**

1 mmol of **4** was dissolved in the suspension of DMF/K_2_CO_3_ (1.1 mmol) and 1 mmol of alkyl halide (iodomethane, iodoethane, propyl bromide and allyl bromide) was added and the mixture was stirred at room temperature until TLC indicated that started material had been consumed (the duration of vortex was controlled to avoid alkylation of the second nitrogen site). Then, the reaction mixture was added to three volumes of cold water and extracted with ethyl acetate. The ethyl acetate extracts were washed with 5% NaOH, water, and dried over Na2SO4. The solvent was removed and the product crystallized in ethanol (yields: 27–54%).

##### 3-Methyl-5-[4-(methylsulfonyl)phenyl]-5-phenylimidazolidine-2,4-dione (N-3-Methyl-5-(4-(methylsulfonyl)phenyl)-5-phenylhydantoin, **5**)

Yield: 29%; mp: 212–214 °C; IR (KBr): ν (cm^−1^) 3230 (NH),1707 (CO),1313, 1153 (SO_2_); ^1^HNMR (CDCl_3_): δ (ppm) 3.09 (s, 3H, CH_3_), 3.15 (s, 3H, SO_2_Me), 6.57 (s, 1H, NH), 7.29–7.44 (m, 5H, Phenyl), 7.69 (d, 2H, 4-methylsufonylphenyl H_2_ & H_6_, *J*=8.4 Hz), 7.98 (d, 2H, 4-methylsufonylphenyl H_3_ & H_5_, *J*=8.4 Hz); 9.42 (s, 1H, NH), 11.23 (s, 1H, NH); ^13^C-NMR (CDCl_3_): δ 29.8, 44.3, 69.4, 126.2, 128.3, 128.5, 129.4, 129.6, 136.5, 139.9, 144.5, 156.7, 163.1; LC-MS (ESI) m/z: 345.1 (M+1) (100); Anal. Calcd. for C_17_H_16_N_2_O_4_S: C, 59.29; H, 4.68; N, 8.13. Found: C, 59.53; H, 4.89; N, 8.01.

##### 3-Ethyl-5-[4-(methylsulfonyl)phenyl]-5-phenylimidazolidine-2,4-dione (N-3-Ethyl-5-(4-(methylsulfonyl)phenyl)-5-phenylhydantoin, **6**)

Yield: 54%; mp: 91–92 °C; IR (KBr): ν (cm^−1^) 3302 (NH),1709 (CO),1310, 1148 (SO_2_); ^1^HNMR (CDCl_3_): δ (ppm) 1.30 (t, 3H, CH_3_, *J*=7.1 Hz), 3.10 (s, 3H, SO_2_Me), 3.70 (q, 2H, CH_2_, *J*=7.1 Hz), 6.79 (s, 1H, NH), 7.29–7.43 (m, 5H, Phenyl), 7.69 (d, 2H, 4-methyl-sufonylphenyl H_2_ & H_6_, *J*=8.4 Hz), 7.98 (d, 2H, 4-methylsufonylphenyl H_3_ & H_5_, *J*=8.4 Hz); ^13^C-NMR (CDCl_3_): δ 12.6, 35.8, 44.2, 69.6, 126.3, 128.3, 128.6, 129.5, 129.7, 136.6, 139.9, 144.5, 156.7, 163.1; LC-MS (ESI) m/z: 359.1 (M+1) (100); Anal. Calcd. for C_18_H_18_N_2_O_4_S: C, 60.32; H, 5.06; N, 7.82. Found: C, 60.12; H, 5.31; N, 8.05.

##### 5-[4-(Methylsulfonyl)phenyl]-5-phenyl-3-propylimidazolidine-2,4-dione (5-(4-(Methylsulfonyl)phenyl)-5-phenyl-N-3-propyl-hydantoin, **7**)

Yield: 47%; mp: 82–83 °C; IR (KBr): ν (cm^−1^) 3302 (NH),1700 (CO),1310, 1150 (SO_2_); ^1^HNMR (CDCl_3_): δ (ppm) 0.95 (t, 3H, CH_3_, *J*=7.4 Hz), 1.72 (m, 2H, CH_2_), 3.09 (s, 3H, SO_2_Me), 3.60 (t, 2H, CH_2_N, *J*=7.3 Hz), 6.38 (s, 1H, NH), 7.29–7.43 (m, 5H, Phenyl), 7.69 (d, 2H, 4-methylsufonylphenyl H_2_ & H_6_, *J*=8.4 Hz), 7.98 (d, 2H, 4-methylsufonylphenyl H_3_ & H_5_, *J*=8.4 Hz); ^13^C-NMR (CDCl_3_): δ 11.2, 19.6, 44.1, 44.4, 69.1, 126.4, 128.3, 128.7, 129.5, 129.7, 136.7, 139.7, 144.7, 156.6, 162.1; LC-MS (ESI) m/z: 373.1 (M+1) (100); Anal. Calcd. for C_19_H_20_N_2_O_4_S: C, 61.27; H, 5.41; N, 7.52. Found: C, 61.52; H, 5.01; N, 7.85.

##### 5-[4-(Methylsulfonyl)phenyl]-5-phenyl-3-(prop-2-en-1-yl)imidazolidine-2,4-dione (N-3-Allyl-5-(4-(methylsulfonyl)phenyl)-5-phenylhydantoin, **8**)

Yield: 27%; mp: 129–130 °C; IR (KBr): ν (cm^−1^) 3322 (NH),1704 (CO),1312, 1152 (SO_2_); ^1^HNMR (CDCl_3_): δ (ppm) 3.10 (s, 3H, SO_2_Me), 4.20 (d, 2H, CH_2_N, *J*=5.6 Hz), 5.22 (d, 2H, CH_2_, *J*=11.3 Hz), 5.85 (m, 1H, CH), 6.55 (s, 1H, NH), 7.29–7.42 (m, 5H, Phenyl), 7.69 (d, 2H, 4-methylsufonylphenyl H_2_ & H_6_, *J*=8.4 Hz), 7.94 (d, 2H, 4-methylsufonylphenyl H_3_ & H_5_, *J*=8.4 Hz); ^13^C-NMR (CDCl_3_): δ 44.1, 69.3, 117.1, 126.3, 128.2, 128.6, 129.4, 129.7, 134.7, 136.8, 139.9, 144.7, 156.8, 161.9; LC-MS (ESI) m/z: 371.1 (M+1) (100); Anal. Calcd. for C_19_H_18_N_2_O_4_S: C, 61.61; H, 4.90; N, 7.56. Found: C, 61.76; H, 5.11; N, 7.25.

### Docking Studies

Docking studies were performed using Autodock software Version 3.0.5. The coordinates of the X-ray crystal structure of the selective COX-2 inhibitor SC-558 bound to the murine COX-2 enzyme was obtained from the RCSB Protein Data Bank (1cx2) and hydrogens were added. The ligand molecules were constructed using the Builder module and were energy minimized for 1000 iterations reaching a convergence of 0.01 kcal/mol Å. The energy minimized ligands were superimposed on SC-558 in the PDB file 1cx2 after which SC-558 was deleted. The purpose of docking is to search for favorable binding configuration between the small flexible ligands and the rigid protein. Protein residues with atoms greater than 7.5 Å from the docking box were removed for efficiency. These docked structures were very similar to the minimized structures obtained initially. The quality of the docked structures was evaluated by measuring the intermolecular energy of the ligand-enzyme assembly [24,25].

### Biological Assay

The ability of the test compounds listed in Table 1 to inhibit ovine COX-1 and COX-2 (IC_50_ value, μM) was determined using chemiluminescent enzyme assays kit (Cayman Chemical, Ann Arbor, MI, USA) according to our previously reported method [23].

## Figures and Tables

**Fig. 1.**
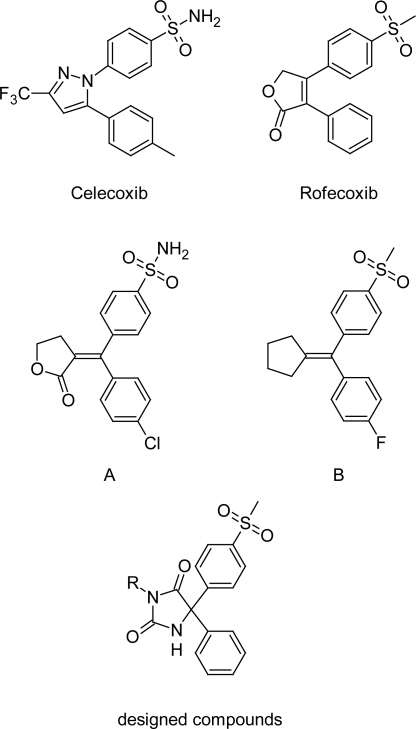
Some representative examples of selective COX-2 inhibitors and our designed compounds.

**Sch. 1.**
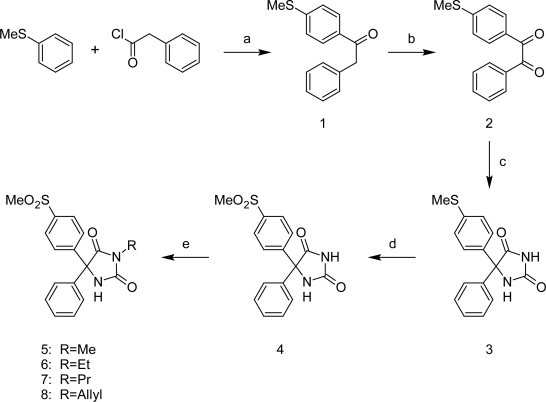
Reagents and conditions: (a) AlCl_3_, CH_2_Cl_2_, 0–25 °C, 2 h (b) SeO_2_, dioxane-H_2_O, reflux, 1 h (c) urea, 30% aqueous NaOH, EtOH, reflux, 3 h (d) oxone, THF-H_2_O, 25 °C, 3 h (e) RI or RBr, K_2_CO_3_/DMF, 10–30 min.

**Tab 1.** In vitro COX-1 and COX-2 enzyme inhibition assay data for 5,5-diarylhydantoin derivatives **4–8**

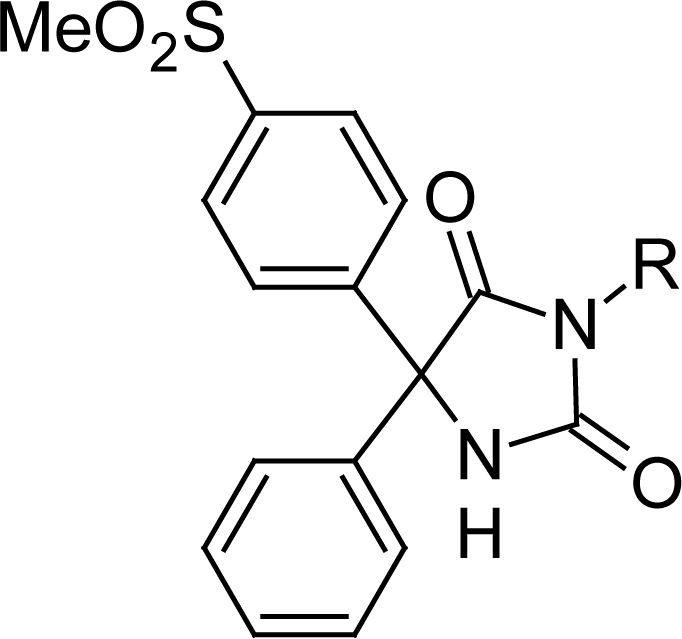				
**Compound**	**R**	**IC_50_** **(μM)^a^**		**Selectivity index (SI)^b^**
		**COX-1**	**COX-2**	

**4**	H	>100	0.077	>1298
**5**	Me	22.69	0.081	280.1
**6**	Et	20.21	0.098	206.2
**7**	Pr	12.01	0.171	70.2
**8**	Allyl	14.96	0.099	151.1
**Celecoxib**	**–**	24.3	0.060	405

a Values are means of two determinations acquired using an ovine COX-1/COX-2 assay kit and the deviation from the mean is < 10% of the mean value.

b In vitro COX-2 selectivity index (COX-1IC_50_/COX-2IC_50_).
